# Evaluating the impact of agricultural abandonment on flood mitigation functions

**DOI:** 10.1038/s41598-025-04419-0

**Published:** 2025-06-12

**Authors:** Takeshi Osawa, Takaaki Nishida, Takashi Oka

**Affiliations:** 1 Graduate School of Urban Environmental Sciences, Tokyo Metropolitan University, Minami-Osawa 1-1, Hachiouji, Tokyo 192-0397 Japan; 2 Kyoto Sangyo University, Motoyama, Kamigamo, Kita-ku, Kyoto 603-8555 Japan; 3 Mitsubishi UFJ Research and Consulting Co. Ltd., 2-5-25, Umeda, Kita-ku, Osaka 530-8213 Japan

**Keywords:** Eco-DRR, Ecosystem service, Floods, Green infrastructure, Seminatural ecosystem, Ecosystem services, Natural hazards

## Abstract

The flood mitigation functions of agricultural ecosystems are crucial in Ecosystem-based Disaster Risk Reduction (Eco-DRR). However, agricultural ecosystems, particularly in developed countries, face increasing abandonment in recent years. This study examined how agricultural abandonment affects Eco-DRR functions in central Japan. In paddy fields, water retention is key for flood mitigation, while in dry farmlands, water infiltration is vital. We analyzed the relationship between abandonment ratios and flood frequency across 132 municipalities in Japan. Results indicated that abandonment had little or no impact on Eco-DRR functions both paddy fields and dry farmlands. For paddy fields, this may be due to high levels of modernization or a low abandonment rate, which can enhance Eco-DRR functions. In dry farmlands, abandonment likely does not affect flood mitigation because it does not impair infiltration functions. Thus, conserving agricultural land is beneficial for Eco-DRR, even if abandoned. Land managers should avoid converting abandoned areas into residential zones or installing artificial structures.

## Introduction

Flooding is one of the most serious natural disasters that affect socioeconomic living^1,2^. 2021 *Disaster in numbers* (2022) reported that Flooding dominated catastrophic events in 2021 at 223 out of 432 total disaster occurrences, which was higher than the average of 163 annual catastrophic floods for 2001–2020^3^. Moreover, their negative impacts have increased over the past 100 years, and several studies have predicted that flooding and the ensuing damage will increase in the future due to climate change, population growth, and economic growth^4–7^. Clearly, the negative impacts of floods must be reduced and strategies aimed at reducing both current and future risks must be developed^8–11^.

Recently, there has been a growing interest in the role of natural or seminatural ecosystems in reducing the frequency and severity of natural disasters, for example ecosystem-based disaster risk reduction [Eco-DRR])^6,10,12–18^. In particular, the Eco-DRR functions of agricultural ecosystems are expected to be globally extensive^15,19,20^. Although there have been a few studies on agricultural ecosystems for Eco-DRR^17^, empirical studies have increased in number recently^9,10,15^. Eco-DRR in agricultural ecosystems is an essential ecosystem service^21^, and natural or seminatural ecosystems that can systematically provide multiecosystem services are expected when applying this idea to green infrastructure^22–24^.

However, agricultural lands, particularly in developed countries, have faced modern problems, particularly with increased the abandonment in recent years^20,25–27^. In this regard, Li and Li (2017) estimated that Western Europe, Southern Europe, North America, and Oceania had increasing abandonment of agricultural areas of 3.79 million km^2^ from 1961 to 2011. Developed countries in Asia have similar trends. In Japan, over 10% of farmland has already been abandoned^26,28^. Although agricultural are expanding ecosystems expand globally, areas of abandoned farmlands are also expanding, especially in developed countries. Therefore, there is one essential question from the perspective of agricultural ecosystems for Eco-DRR: How does agricultural abandonment influence Eco-DRR function?^10^.

The change in the Eco-DRR function by abandonment is complex, and these effects differ between paddy fields and dry farmlands. The flooding mitigation function in agricultural ecosystems is supported by their water retention capacity and the infiltration of water^29,30^. The former function is expected in paddy fields^31,32^. This function is strongly influenced by ditch–ridge structures, which could collapse due to abandonment^33^; thus, abandonment could negatively influence the Eco-DRR function in paddy fields. Infiltration is mainly expected in dry farmlands^34^. In dry farmlands, once abandonment occurs, runoff water and sediment concentrations suddenly increase but not for long periods^35^. This result suggests that abandonment could negatively influence the Eco-DRR function only for a short time in dry farmlands. The infiltration function is strongly influenced by agricultural practices, because it is regulated by vegetation and soil conditions^30^. After abandonment, the soil conditions^36,37^ and vegetation structures^10,28^ in farmlands change drastically; thus, the infiltration function can also change. However, abandonment can positively influence the Eco-DRR function, as previous studies have indicated that soil porosity increased in abandoned dry farmlands^37^ and vegetation recovery occurred^38^.

Here, we investigated the relationship between agricultural abandonment and the Eco-DRR function at large geographical scales in central Japan to understand their general patterns. We studied paddy fields and dry farmlands separately because of the different mechanisms in terms of specific water retention in paddy fields and infiltration in dry farmlands, where abandonment can cause different environmental changes. In addition, we considered the local conditions of agricultural land, as previous studies have suggested that the flood mitigation function of agricultural land is strongly restricted by location, not restricted by their area size ^9,10,15^. We predicted that the effects of abandonment on the Eco-DRR function would differ between paddy fields and dry farmlands because of the different types of conditional change after abandonment. Specifically, the Eco-DRR function in abandoned paddy fields could decline, whereas that in dry farmlands could have no notable decline. Based on the results, we discuss the effects of flood DRR by agricultural abandonment and the appropriate management thereof.

## Results

**Fig. 1:**
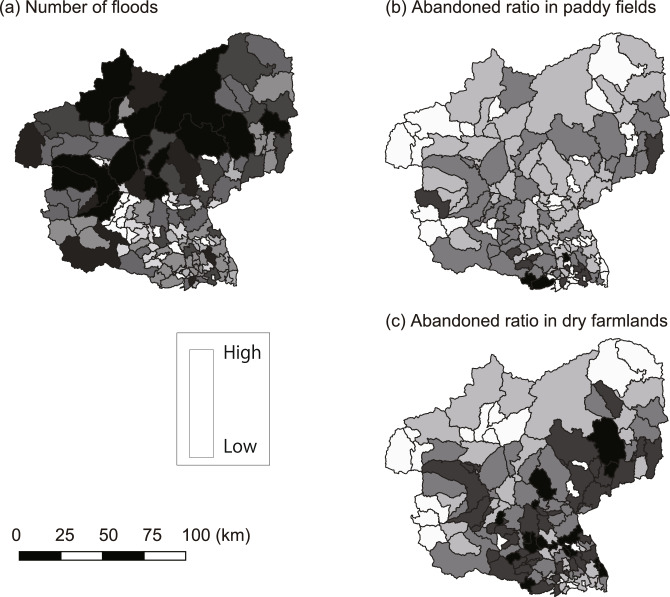
Maps on (**a**) flood frequency from 2011 to 2019, (**b**) abandonment ratio in paddy fields, and (**c**) abandonment ratio in dry farmlands in the study region. The gradient values are not standardized, they represent relative values within each of (**a**), (**b**), and (**c**), and the absolute values differ among these.

All the variables for the analysis in each municipality are listed in Table 1. There were 5.2 ± 2.1 (mean ± standard deviation [SD]) floods from 2011 to 2019 in various municipalities (Table 1). Flood frequency was relatively high in the northern area of the study region (Fig. 1). During the study period, nine municipalities experienced annual flood events (Appendix Table 1). From 2011 to 2019, nine municipalities were merged through governmental policies (Appendix Table 1).

**Table 1:** Summary of variable for eash municipality.

Variables	Mean ± S.D
Number of floods	5.2 ± 2.1
FAV in tital paddy field	1,625,746 ± 3,174,924
FAV in active paddy field	1,542,368 ± 3,018 ,966
The ratio of FAV in abandoned paddy field	0.06 ± 0.07
FAV in total dry farmland	422,645 ± 668,660
FAV in active dry farmland	351,405 ± 561,286
The ratio of FAV in abandoned dry farmland	0.18 ± 0.09
FAV un urban area	968,838 ± 1,600,816
Area of the municipality (km^2^)	124.9 ± 180.7

On the paddy fields, the total abandonment ratio from the CAFF data was 0.05, and the average value from the studied municipalities was 0.06 ± 0.07 (Average ± SD) (Table 1). Thus, approximately 95% of FAVs in the total paddy fields were active paddy fields (1,542,368/1,625,746). The abandonment ratio of the paddy fields was generally consistent across the study region (Fig. 1). Meanwhile, the total abandonment ratio from the CAFF data was 0.15 for the dry farmlands, and the average value in the studied municipalities was 0.18 ± 0.09 (Table 1). Thus, approximately 83% of FAVs in the total dry farmlands were in active dry farmlands (351,405/422,645). The FAV in the urban areas was 968,838 ± 1,600,816 (Table 1). The abandonment ratio of dry farmlands was relatively high in the central and southern areas of the study region (Fig. 1). The area of each municipalities was 124.9 ± 180.7 km^2^ (Table 1).

The GLMM analyses for the number of floods for each municipality focusing on paddy fields showed that the FAV in total paddy fields (Model 1 and 3) and the FAV in active paddy fields (Model 2 and 4) were negatively correlated with flood frequency, whereas the FAV in urban areas and the area of the municipality were positively correlated (Model 1–4) (Table 2). The FAV ratio in abandoned paddy fields was not correlated with the number of floods (Model 3 and 4) (Table 2). Among the four models, the AIC was basically the same, with differences within 2, which shows that the model performances were basically the same (Table 2).

**Table 2:** GLMM results for the frequency of floods in the paddy fields.

Explanatory variables	Paddy model 1 (S.D.)	p	Paddy model 2 (S.D.)	p	Paddy model 3 (S.D.)	p	Paddy model 4 (S.D.)	p
FAV in total paddy field	−0.02	*	–		−0.02	*	–	
	(0.01)				(0.01)			
FAV in active paddy field	–		−0.02	*	–		−0.02	
			(0.01)				(0.01)	
FAV in urban area	0.07	*	0.07	*	0.07	*	0.07	*
	(0.03)		(0.03)		(0.03)		(0.03)	
Area of municipal	0.22	***	0.22	***	0.22	***	0.22	***
	(0.04)		(0.04)		(0.04)		(0.04)	
Ratio of FAV in abandonment	–		–		0.38	n.s.	0.36	n.s.
					(0.59)		(0.59)	
Intercept	−3.16		−3.17		−3.21		–3.21	
	(0.66)		(0.66)		(0.67)		(0.67)	
AIC	532.42		532.37		534.01		534.01	

Results of wald test, *: p< 0.05: **:p <0.01, ***:p <0.001.

The GLMM analyses for the number of floods in each municipality, focusing on dry farmlands, showed the same trends as those for paddy fields. The FAV in total dry farmlands (Model 1 and 3) and the FAV in active dry farmlands (Model 2 and 4) were negatively correlated, whereas the FAV in urban areas and the municipality were positively correlated (Model 1–4) (Table 3). The FAV ratio in abandoned dry farmlands had no correlation with the number of floods (Model 3 and 4) (Table 3). As with the paddy field models, among the four models for dry farmlands, the AIC was basically the same, with differences at least within 2, which shows that the model performances were basically the same (Table 2). Thus, both paddy fields and dry farmlands, including abandoned lands, can mitigate the occurrence of floods, whereas urbanized areas enhance the occurrence of floods. Agricultural abandonment did not strongly influence the Eco-DRR function in either paddy fields or dry farmlands.

**Table 3:** GLMM results for the frequency of floods in the dry farmlands.

Explanatory variables	Farmland model 1	p	Farmland model 2	p	Farmland model 3	p	Farmland model 4	p
FAV in farmland	−0.06	**	–		−0.06	**	–	
	(0.02)				(0.02)			
FAV in active farmland	–		−0.06	**	–		−0.06	**
			(0.02)				(0.02)	
FAV in urban	0.07	*	0.07	*	0.07		0.07	*
	(0.03)		(0.03)		(0.03)		(0.03)	
Area of municipal	0.27	***	0.27	***	0.28	***	0.28	***
	(0.04)		(0.04)		(0.05)		(0.05)	
Ratio of FAV in abandonment	–		–		0.15	n.s.	0.08	n.s.
					(0.44)		(0.43)	
Intercept	−3.55		−3.58		−3.62		−3.62	
	(0.68)		(0.68)		(0.71)		(0.71)	
AIC	526.12		526.02		528		527.99	

Results of wald test, *: p< 0.05: **:p <0.01, ***:p <0.001.

## Discussion

This study tested the relationship between agricultural abandonment and flood frequency by studying paddy fields and dry farmlands separately in the central region of Japan. Although we predicted that the effects of abandonment are different between paddy fields and dry farmlands, the results were basically the same. Statistical modeling showed that the abandonment of both paddy fields and dry farmlands had little or no effect on the Eco-DRR function in the studied area.

Paddy fields inherently have a water-retaining function because they are agricultural lands designed for farming artificial wetlands by storing water. Therefore, they are anticipated to serve as reservoirs for rainwater and floodwater from the perspective of Eco-DRR^15,21^. This function is strongly influenced not only by the condition of the paddy surface itself but also by its facilities such as ditches^31^. Generally, after abandonment, the maintenance of these facilities is neglected, which could lead to a decline in the water-retaining function of the paddy field^33^. Therefore, we predicted that abandonment would negatively influence the Eco-DRR function in paddy fields. However, our results showed that abandoned paddy fields did not negatively influence the frequency of floods. A possible explanation for this result is modernization. Recently, over half of the agricultural lands have been modernized, including those concreted for ditch–ridge structures^39,40^. Although sometimes neglected upon abandonment, concrete ditch–ridge structures can retain their retaining function because concrete facilities are more durable and can better maintain their water retention capabilities than traditional soil facilities. Evaluation of this possibility is the next challenge for detailed field surveys. Another possible explanation is the size effect. In the study area, approximately 5% of the paddy fields were abandoned. This ratio is relatively small compared to the total abandonment ratio in Japan, which is currently over 10% currently (Ministry of Agriculture, Forestry and Fisheries, Japan, https://www.maff.go.jp/j/study/nouti_seisaku/senmon_04/pdf/data6.pdf, accessed May 14, 2025). In addition, FAV, which is an essential indicator of the Eco-DRR function in paddy fields^9,10,15^, showed a small difference between the total paddy fields and the active paddy fields. Thus, the study area was too small to show an evident decline in the Eco-DRR function. Previous studies have suggested that paddy fields with high FAV values are often large in area and have undergone efficiency improvements such as mechanization^41^. Consequently, it is possible that paddy fields with lower Eco-DRR functionality are more likely to be abandoned. To test this is challenging within the framework of the present study, which focuses on identifying common trends at the municipal level. Therefore, further investigation at a finer spatial scale is necessary.

Dry farmlands are typically covered with fertile soils and crops. Consequently, from the perspective of Eco-DRR, they are anticipated to have a significant water infiltration function^34^. Our results suggest that abandonment of dry farmlands does not negatively influence the frequency of floods. The infiltration function of dry farmlands is mainly regulated by vegetation and soil conditions^30^. In abandoned dry farmlands, vegetation other than crops can grow; thus, soil can also be maintained. This suggests that the infiltration capacity may not necessarily decline in abandoned dry farmlands. In the study area, approximately 15% of dry farmland was abandoned, which is a relatively large area compared with the total abandonment ratio in Japan (Ministry of Agriculture, Forestry and Fisheries, Japan, https://www.maff.go.jp/j/study/nouti_seisaku/senmon_04/pdf/data6.pdf, accessed May 14, 2025). Nevertheless, our results demonstrate that abandoned dry farmlands did not negatively influence the frequency of floods in this study. According to these findings, the abandonment of dry farmlands may not significantly impact their infiltration capacity, which is related to Eco-DRR functions. However, changes in both vegetation and soil conditions after abandonment might vary depending on the soil properties, locations, and climatic factors. Additionally, the time elapsed after abandonment might be essential. Evaluating these differences among abandoned dry farmlands are worth testing in future studies.

In both paddy field and dry farmland models, the FAV in urbanized areas positively correlated with flooding frequency. This result matched that of a previous studies^9,42,43^ and was reasonable because high-FAV areas converged water from the surrounding areas. Thus, these areas tend to have concentrated runoff water, which could cause flooding^9,15^. Areas that tend to concentrate water in urban areas enhance the occurrence of flooding damage^9,42^. Therefore, our statistical models were reasonable. However, it should be noted that the statistical modeling approach used in this study does not explicitly capture hydrological processes, such as water retention in paddy fields or infiltration in upland fields, which are assumed mechanisms in our study. Furthermore, because the occurrence of flood damage is strongly influenced by probabilistic events such as localized heavy rainfall^43^, it is important to interpret the results as a pattern-based analysis conducted at the municipal level, rather than as a direct representation of hydrological dynamics.

This study tested the relationship between flood occurrences and agricultural abandonment, mainly based on governmental statistics, which were considered effective in evaluating the study aim. However, several issues remain to be addressed. The limitations of the data used in this study should be acknowledged. The statistical information employed indicates only the presence or absence of flood damage within a given year, and does not include data on the specific timing of flood events. In the case of paddy fields, which are typically filled with water during the cultivation season, if their disaster mitigation capacity varies depending on whether they are inundated at the time of flooding, this could potentially lead to an underestimation of the impact of abandonment. However, even during the cultivation period, paddy fields are not consistently filled to capacity; thus, it is unlikely that their flood retention capacity would be drastically reduced. Therefore, we believe that this limitation did not substantially affect the overall results. Another point of consideration is the nature of flood events classified as damage in the statistical data. The disaster risk reduction functions of agricultural land assessed in this study are primarily based on two mechanisms: reducing the runoff of rainwater into rivers, and absorbing overflow from rivers. However, some of the recorded flood events may include those caused by structural failures such as dam collapses or levee breaches. However, such structural failures are relatively rare occurrences, and we consider it unlikely that they significantly influenced the results of this study. These limitations are inherent to the research approach, which aims to analyze common patterns using statistical data. Future studies should evaluate the effects of agricultural land abandonment at finer spatial scales and resolutions to capture its impact more accurately.

## Conclusion

This study suggests that both abandoned paddy fields and abandoned dry farmlands could maintain the function of flood mitigation to at least some degree. Thus, the conservation of agricultural land with water storage zones is effective for the Eco-DRR, even for abandoned agricultural areas. Therefore, converting these areas into residential areas, installing artificial structures, or any other modifications that might diminish Eco-DRR functions should be avoided. However, maintaining agricultural land that has already lost its essential function (i.e., food production) is not straightforward. For abandoned farmlands with high Eco-DRR functions, policy support such as providing subsidies for their maintenance might be necessary. Meanwhile, in recent years, the concept of “livelihood resilience” has been increasingly discussed, referring to the capacity of individuals or households to cope with environmental changes by adapting their livelihood strategies^44,45^. Given the global expansion of abandoned agricultural land, it may be necessary to consider maintaining livelihoods for landowners^46^ not only to maintain such land for agricultural use at all costs but also to fundamentally rethink land use, such as repurposing it for disaster risk reduction with payment through public funds as a viable alternative.

## Methods

### Study region

**Fig. 2:**
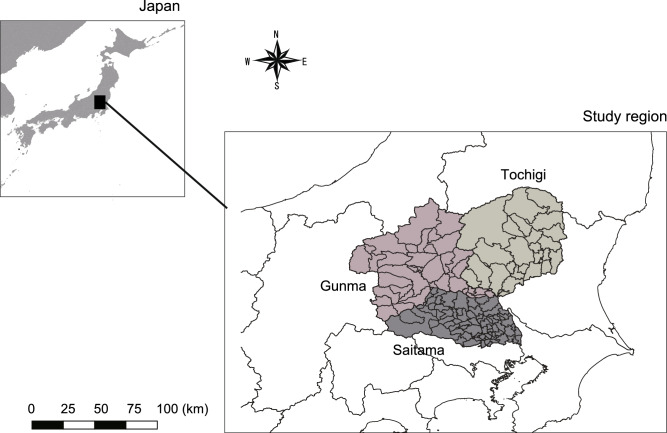
Location of three prefectures: Saitama, Tochigi, and Gunma.

This research studied 132 municipalities in Saitama, Tochigi, and Gunma Prefectures in Japan (Fig. 2). They were chosen because they do not border the sea and are therefore unaffected by damage from coastal floods and tsunamis, allowing the study to focus only on land-based flood disasters. Also, the proportion of the total area of agricultural land is relatively high, with Saitama Prefecture at 19.5%, Tochigi Prefecture at 19.0% and Gunma Prefecture at 10.5%, respectively (https://nlab.itmedia.co.jp/research/articles/469457/2, https://www.e-stat.go.jp/stat-search/files?page=1amp:layout=datalistamp:toukei=00500215amp:tstat=000001013427amp:cycle=7amp:year=20200amp:month=0amp:tclass1=000001032270amp:tclass2=000001032271amp:tclass3=000001150346amp:tclass4val=0, accessed May 14, 2025). In contrast, the neighboring prefectures which do not border the sea of Nagano and Yamanashi exhibited lower farmland ratios of 7.8% and 5.2%, respectively.

### Flood and natural disaster data

Flood statistics recorded by the Japanese government from 1961 to the present are available. From these records, we extracted digitized data between 2011 and 2019 available online at the study term from Ministry of Land, Infrastructure, Transport and Tourism (MLIT) portal (https://www.e-stat.go.jp/stat-search/files?page=1&toukei=00600590&result_page=1, accessed August 23, 2023), which suits land cover and agricultural abandonment data (described below). These statistical data covered the areas that had suffered floods, number of damaged buildings, and cost per year in each municipality, regardless of their scale, that resulted in human and economic damage to assets and other properties (https://www.e-stat.go.jp/stat-search/files?page=1&toukei=00600590amp:result_page=1, accessed March 1, 2025). Therefore, the occurrence of flooding did not indicate an overflow phenomenon but the damage caused by an overflow. If there was no flood damage to the municipality in a year, the values were set to 0. Using this dataset, the number of floods in each municipality from 2011 to 2019 was determined. Therefore, the maximum number of floods was 9, and the minimum was 0. Before the analysis, we excluded municipalities that were merged through governmental policies.

### Land cover and terrain data

We prepared a digitized land-use/land-cover map with land utilization statistics represented by segmented mesh data in 2016, which matched the flood statistics (2011–2019) derived from the National Land Numerical Information Survey (https://nlftp.mlit.go.jp/ksj/gml/datalist/KsjTmplt-L03-b.html, accessed May 14, 2025). Land-use/land-cover data were developed using both topographic maps and satellite imaging, with 12 land-use classifications based on nationwide land utilization with a 100-m grid resolution: paddy fields, other agricultural lands, forests, wastelands, urbanized areas, roads, railways, other artificial land use, rivers and ponds, seashores, seas, and golf fields (National Land Information Division, MLIT: https://nlftp.mlit.go.jp/ksj/gml/codelist/LandUseCd-09.html, accessed May 14, 2025). In the National Land Numerical Information Survey, “other agricultural land” is defined as land used for cultivating crops such as wheat, upland rice, vegetables, grass, turf, apples, pears, peaches, grapes, tea, paulownia, Japanese wax tree, paper mulberry, and windmill palm (https://nlftp.mlit.go.jp/ksj/gml/codelist/LandUseCd-09.html, accessed May 14, 2025). Based on this definition, it can be interpreted to include both dry upland fields and orchards. Therefore, we used paddy fields, other agricultural lands as dry farmlands, and urban area data for the analysis. We included the urban areas in this study because previous studies indicated that the extent of urban land within a municipality influences the occurrence of flooding^9,43^.

We used the flow accumulation value (FAV), which refers to the sum of the weights of all cells flowing into each downslope cell, to define concave areas (ESRI; https://pro.arcgis.com/ja/pro-app/tool-reference/spatial-analyst/how-flow-accumulation-works.htm, accessed August 23, 2023). Lower elevations and valley areas had higher FAVs because they could store larger quantities of water, whereas higher ridge areas had lower FAVs. Previous studies have demonstrated that the FAV in such locations could reflect Eco-DRR functions, and the area size could not reflect them^9,10,15^. Thus, we calculated the FAV for the entire mainland of Japan; this range could cover the entire basin that overlapped with our target areas. The FAVs were computed using ArcGIS version 10.5 with Spatial Analyst (ESRI, Redlands, CA, USA) using a 50-m digital elevation model sourced from the Japanese Map Center (http://www.jmc.or.jp/, accessed August 23, 2023). After calculating the FAVs for each 50-m grid in each municipality, we overlaid land-use maps, which had three land categories. The FAVs were then summed for each land use to determine the potential Eco-DRR function.

### Agricultural abandonment data

We obtained conditional agricultural land data from the Census for Agriculture, Forestry and Fisheries (CAFF) dataset (https://www.maff.go.jp/j/tokei/census/afc/, August 23, 2023). This census was conducted every 5 years by the Ministry of Agriculture, Forestry and Fisheries of Japan. We used data released in 2015, which matched the years on the land-use map in 2016. The CAFF dataset includes the area of total agricultural land, paddy fields, and dry farmlands and those abandoned in each agricultural land type in each municipality. Using CAFF data, we calculated the ratio of abandonment in both paddy fields and dry farmlands in each municipality. Thus, we obtained the abandonment ratios for each land type in each municipality. We then multiplied the ratios of abandonment from the CAFF data and FAV by the agricultural land from the land-use map in each municipality. The obtained value indicated the FAV of the abandoned agricultural land in each type. The total FAV in agricultural land minus the FAV in abandoned agricultural land indicated the FAV in operating (i.e., active, nonabandoned) agricultural land. We calculated these values for both paddy fields and dry farmland in each municipality.

### Statistical analysis

The number of floods from 2011 to 2019 in each municipality was tested using a generalized linear mixed-effects model (GLMM) with a Poisson distribution (log link) and Wald test. To analyze the paddy fields, we used four combinations of explanatory variables: (1) FAV in total paddy fields, FAV in urban areas, and area of the municipality; (2) FAV in active paddy fields (excluding abandoned paddy fields), FAV in urban areas, and area of the municipality; (3) FAV in total paddy fields, the ratio of FAV in abandoned paddy fields within the total FAV in paddy fields, FAV in urban areas, and area of the municipality; and (4) FAV in active paddy fields (excluding abandoned paddy fields), the ratio of FAV in abandoned paddy fields within the total FAV in paddy fields, FAV in urban areas, and area of the municipality. We decided these explanatory variables i.e., FAV in agricultural land and urban areas according to the previsou study^9^. We calculated the Akaike’s information criterion (AIC) to compare the fittings among the four models. We used municipality ID, i.e., a unique identifier for each municipality, as a random term in this model. All explanatory variables, excluding the ratio of FAV in abandoned paddy fields to the total FAV in paddy fields, were log-transformed.

Similarly, to analyze dry farmland, we constructed statistical models using the same four combinations of explanatory variables as those for paddy fields, substituting the paddy field variable with dry farmlands. The analytical method and treatment of the explanatory variables were identical to those used in paddy field analysis.

Before the analysis, we tested all combinations of the explanatory variables for multicollinearity by calculating variance inflation factors (VIFs)^47^ using the R package “car.” We observed no significant multicollinearity (VIF < 2 for all variable combinations). We could not combine the FAVs in both paddy fields and dry farmlands, because the VIF increased significantly when we included these variables concurrently.

## Supplementary Information

Supplementary Information.

## Data Availability

All datasets are freely available on the website shown in the text body.

## References

[1] Istomina, M. N. & Dobrovoski, S. G. Floods of the world: Quantitative analysis of natural characteristics and parameters of social-economic damages. *Water Resour.* **43**, 459–471 (2016).

[2] Rehman, J., Sohaib, O., Asif, M. & Pradhan, B. Applying systems thinking to flood disaster management for a sustainable development. *Int. J. Disaster Risk Red.* **36**, 101101 (2019).

[3] 2021 Disasters in numbers - World | ReliefWeb. https://reliefweb.int/report/world/2021-disasters-numbers. (2022).

[4] Ahmad, S. S. & Simonovic, S. P. A three-dimensional fuzzy methodology for flood risk analysis. *J. Flood Risk Manag.* **4**, 53–74 (2011).

[5] Arnell, N. W. & Gosling, S. N. The impacts of climate change on river flood risk at the global scale. *Clim. Chang.* **134**, 387–401 (2016).

[6] Nakamura, F. et al. Adaptation to climate change and conservation of biodiversity using green infrastructure. *River Res. Appl.*10.1002/rra.3576 (2019).

[7] Ward, P. J. et al. Review article: Natural hazard risk assessments at the global scale. *Nat. Hazard.* **20**, 1069–1096 (2020).

[8] Akasaka, T. et al. Reconciling biodiversity conservation and flood risk reduction : The new strategy for freshwater protected areas. *Divers. Distrib.*10.1111/ddi.13517 (2022).

[9] Osawa, T. Evaluating the effectiveness of basin management using agricultural land for ecosystem-based disaster risk reduction. *Int. J. Disaster Risk Red.* **83**, 103445 (2022).

[10] Osawa, T., Nishida, T. & Oka, T. Potential of mitigating floodwater damage to residential areas using paddy fields in water storage zones. *Int. J. Disaster Risk Red.* **62**, 102410 (2021).

[11] Ward, P. J. et al. The need to integrate flood and drought disaster risk reduction strategies. *Water Secur.* **11**, 100070 (2020).

[12] Furuta, N. & Shimatani, Y. Integrating ecological perspectives into engineering practices—Perspectives and lessons from Japan. *Int. J. Disaster Risk Red.*10.1016/j.ijdrr.2017.12.003 (2017).

[13] Martin, T. G. & Watson, J. E. M. Intact ecosystems provide best defence against climate change. *Nat. Clim. Chang.* **6**, 122–124 (2016).

[14] Mori, S., Pacetti, T., Brandimarte, L., Santolini, R. & Caporali, E. A methodology for assessing spatio-temporal dynamics of flood regulating services. *Ecol. Ind.* **129**, 107963 (2021).

[15] Osawa, T., Nishida, T. & Oka, T. High tolerance land use against flood disasters: How paddy fields as previously natural wetland inhibit the occurrence of floods. *Ecol. Ind.* **114**, 106306 (2020).

[16] Scarano, F. R. Ecosystem-based adaptation to climate change: concept, scalability and a role for conservation science. *Perspect. Ecol. Conserv.* **15**, 65–73 (2017).

[17] Sudmeier-Rieux, K. et al. Scientific evidence for ecosystem-based disaster risk reduction. *Nat Sustain* **4**, 803–810 (2021).

[18] Wickramasinghe, D. Ecosystem-based disaster risk reduction. *Oxford Res. Encycl. Nat. Hazard Sci.*10.1093/acrefore/9780199389407.013.360 (2021).

[19] Foley, J. A. et al. Global consequences of land use. *Science* **309**, 570–574 (2005). 16040698 10.1126/science.1111772

[20] Winkler, K., Fuchs, R., Rounsevell, M. & Herold, M. Global land use changes are four times greater than previously estimated. *Nat. Commun.* **12**, 2501 (2021). 33976120 10.1038/s41467-021-22702-2 PMC8113269

[21] Natuhara, Y. Ecosystem services by paddy fields as substitutes of natural wetlands in Japan. *Ecol. Eng.* **56**, 97–106 (2013).

[22] Madureira, H. & Andresen, T. Planning for multifunctional urban green infrastructures: Promises and challenges. *Urban Des. Int.* **19**, 38–49 (2014).

[23] Osawa, T., Nishida, T. & Oka, T. Paddy fields as green infrastructure: their ecosystem services and threatening drivers. In *Green Infrastructure and Climate Change Adaptation* (ed. Nakamura, F.) 175–185 (Springer, 2022).

[24] Roe, M. & Mell, I. Negotiating value and priorities: evaluating the demands of green infrastructure development. *J. Environ. Plan. Manag.* **56**, 650–673 (2013).

[25] Li, S. & Li, X. Global understanding of farmland abandonment: A review and prospects. *J. Geogr. Sci.* **27**, 1123–1150 (2017).

[26] Osawa, T., Kohyama, K. & Mitsuhashi, H. Multiple factors drive regional agricultural abandonment. *Sci. Total Environ.* **542**, 478–483 (2016). 26520271 10.1016/j.scitotenv.2015.10.067

[27] Ustaoglu, E. & Collier, M. J. Farmland abandonment in Europe: an overview of drivers, consequences, and assessment of the sustainability implications. *Environ. Rev.* **26**, 396–416 (2018).

[28] Osawa, T., Kohyama, K. & Mitsuhashi, H. Areas of increasing agricultural abandonment overlap the distribution of previously common, currently threatened plant species. *PLoS ONE* **8**, e79978 (2013). 24260328 10.1371/journal.pone.0079978 PMC3832657

[29] Barral, M. P., Laterra, P. & Maceira, N. Flood mitigation ecosystem service in landscapes of Argentina’s Pampas: identifying winning and losing farmers. *J. Environ. Manag.* **240**, 168–176 (2019). 10.1016/j.jenvman.2019.03.099 30933821

[30] O’Connell, P. E., Ewen, J., O’Donnell, G. & Quinn, P. Is there a link between agricultural land-use management and flooding?. *Hydrol. Earth Syst. Sci.* **11**, 96–107 (2007).

[31] Suzuki, Y., Nakamura, K. & Hama, T. Peak discharge mitigation effects in different rainfall patterns at a paddy plot with a runoff control plate. *J. Hydrol. Reg. Stud.* **42**, 101165 (2022).

[32] Yoshikawa, N., Nagao, N. & Misawa, S. Evaluation of the flood mitigation effect of a paddy field dam project. *Agric. Water Manag.* **97**, 259–270 (2010).

[33] Imai, Y., Muto, Y. & Kamada, M. Change in floodwater retention function of a paddy field due to cultivation abandonment in a depopulating rural region in Japan. In *Green Infrastructure and Climate Change Adaptation: Function, Implementation and Governance* (ed. Nakamura, F.) 161–173 (Springer Nature, 2022).

[34] Bachand, P. et al. On-farm flood capture could reduce groundwater overdraft in Kings River Basin. *Calif. Agric.* **70**, 200–207 (2016).

[35] Cerdà, A. et al. Long-term impact of rainfed agricultural land abandonment on soil erosion in the Western Mediterranean basin. *Prog. Phys. Geogr. Earth Environ.* **42**, 202–219 (2018).

[36] Dunjó, G., Pardini, G. & Gispert, M. Land use change effects on abandoned terraced soils in a Mediterranean catchment NE Spain. *CATENA* **52**, 23–37 (2003).

[37] Raiesi, F. Soil properties and C dynamics in abandoned and cultivated farmlands in a semi-arid ecosystem. *Plant Soil* **351**, 161–175 (2012).

[38] García-Ruiz, J. M. & Lana-Renault, N. Hydrological and erosive consequences of farmland abandonment in Europe, with special reference to the Mediterranean region—A review. *Agr. Ecosyst. Environ.* **140**, 317–338 (2011).

[39] Katayama, N., Baba, Y. G., Kusumoto, Y. & Tanaka, K. A review of post-war changes in rice farming and biodiversity in Japan. *Agric. Syst.* **132**, 73–84 (2015).

[40] Osawa, T., Kohyama, K. & Mitsuhashi, H. Trade-off relationship between modern agriculture and biodiversity: Heavy consolidation work has a long-term negative impact on plant species diversity. *Land Use Policy* **54**, 78–84 (2016).

[41] Osawa, T. Overlap relationship between the priority of land consolidation and the floodplain wetland potential in paddy field. *Ecol. Res.* **39**, 242–249 (2024).

[42] Osawa, T. Agricultural land around river confluences could strongly suppress floods occurrences. *Environ. Sustain. Indic.* **24**, 100533 (2024).

[43] Osawa, T., Sakurai, G. & Wakai, A. Developing national-scale basic guideline on flood-adaptation strategies under climate change using probabilistic and deterministic factors. *Water Res.* **282**, 123723 (2025). 40319782 10.1016/j.watres.2025.123723

[44] Liu, W., Li, J. & Xu, J. Effects of disaster-related resettlement on the livelihood resilience of rural households in China. *Int. J. Disaster Risk Red.* **49**, 101649 (2020).

[45] Li, T. et al. Livelihood resilience in pastoral communities: Methodological and field insights from Qinghai-Tibetan Plateau. *Sci. Total Environ.* **838**, 155960 (2022). 35588815 10.1016/j.scitotenv.2022.155960

[46] Li, T. et al. Navigating the landscape of global sustainable livelihood research: past insights and future trajectory. *Environ. Sci. Pollut. Res.* **30**, 103291–103312 (2023). 10.1007/s11356-023-29567-6 37684508

[47] Dormann, C. F. et al. Collinearity: A review of methods to deal with it and a simulation study evaluating their performance. *Ecography* **36**, 027–046 (2013).

